# A phase 1 study in healthy participants to characterize the safety and pharmacology of inclacumab, a fully human anti-P-selectin antibody, in development for treatment of sickle cell disease

**DOI:** 10.1007/s00228-023-03514-3

**Published:** 2023-07-12

**Authors:** Christina Lourdes Mayer, Kathleen Koeck, Margot Hottmann, Andrew Redfern, Mark Davis, Aline Barth, Xin Geng, Carolyn Hoppe, Patrick Yue

**Affiliations:** 1 Semivida Research, Dallas, TX USA; 2 Certara USA, Inc., Princeton, NJ USA; 3 Pfizer Inc, New York, NY USA; 4 Linear Clinical Research, Nedlands, WA Australia

**Keywords:** Inclacumab, P-selectin, Monoclonal antibody, Pharmacokinetics, Pharmacodynamics, Sickle cell disease

## Abstract

**Purpose:**

We evaluated the safety, pharmacokinetics (PK), pharmacodynamics (PD), and immunogenicity of intravenous (IV) inclacumab, a fully human IgG4 anti–P-selectin monoclonal antibody in development for the treatment of sickle cell disease, at doses up to and exceeding those previously tested in healthy individuals.

**Methods:**

In this phase 1, open-label, single-ascending-dose study, 15 healthy participants were enrolled into cohorts receiving 20 mg/kg (*n* = 6) or 40 mg/kg (*n* = 9) IV inclacumab and observed for up to 29 weeks post-dose. Safety, PK parameters, thrombin receptor-activating peptide (TRAP)-activated platelet-leukocyte aggregate (PLA) formation, P-selectin inhibition, plasma soluble P-selectin, and anti-drug antibodies were characterized.

**Results:**

Two inclacumab-related treatment-emergent adverse events were reported in 1 participant; no dose-limiting toxicities were observed. Plasma PK parameters were generally dose-proportional, with a terminal half-life of 13 to 17 days. Mean TRAP-activated PLA formation decreased within 3 h from the start of infusion, and inhibition was sustained for ~ 23 weeks. Mean P-selectin inhibition > 90% was observed up to 12 weeks post-dose. The mean ratio of free to total soluble P-selectin decreased rapidly from pre-dose to end of infusion, then increased gradually to 78% of the baseline ratio by week 29. Treatment-emergent anti-drug antibodies were observed in 2 of 15 participants (13%), without apparent impact on safety, PK, or PD.

**Conclusions:**

Inclacumab was well tolerated, with PK as expected for a monoclonal antibody against a membrane-bound target and a long duration of PD effects after both single IV doses, supporting a prolonged dosing interval.

**Trial registration:**

ACTRN12620001156976; registered November 4, 2020.

## Introduction

P-selectin is a cell adhesion molecule expressed by platelets and endothelial cells [1]. When these cells are activated in response to vascular injury and/or inflammation (e.g., by hypoxia, heme, complement components, thrombin, or histamines), P-selectin is translocated to the cell surface where it can bind to its primary ligand, P-selectin glycoprotein ligand 1 (PSGL-1), and thereby mediate leukocyte recruitment by platelets and endothelial cells [1, 2]. As such, P-selectin plays an essential role in facilitating the intercellular interactions central to thrombotic and inflammatory processes [1, 2].

In sickle cell disease (SCD), polymerization of sickle hemoglobin leads to rigid, sticky, sickled red blood cells (RBCs) that are prone to hemolysis, creating a procoagulant and proinflammatory environment that contributes to vaso-occlusive crises (VOCs), one of the clinical hallmarks of the disease [3–5]. VOCs are triggered by the adhesion of leukocytes, platelets, and sickle RBCs to the endothelium of blood vessels, causing vascular obstruction and consequent tissue ischemia that manifests as pain and, in some cases, can cause death [3, 6]. By mediating platelet-leukocyte aggregate (PLA) formation and leukocyte adhesion to the vascular endothelium, P-selectin is known to contribute to the occurrence of VOCs in SCD [3, 7]. Blocking this interaction may reduce the frequency of VOCs in patients with SCD [7, 8].

Inclacumab is an investigational mAb that is also directed against human P-selectin [9]; however, it is a fully human mAb that targets the PSGL-1 binding region of P-selectin with high affinity [9, 10]. In a preclinical study, inclacumab showed greater maximal inhibition of cell-cell interactions than crizanlizumab [9]. In addition, previous clinical studies have shown that a single intravenous (IV) dose of inclacumab 20 mg/kg was able to sustain PLA inhibition for at least 12 weeks in healthy individuals, suggesting that a less frequent, and therefore more convenient, dosing interval may be possible [9–11].

Here, we report results from a phase 1, open-label, single-ascending-dose study of inclacumab designed to characterize the safety, tolerability, pharmacokinetics (PK), pharmacodynamics (PD), and immunogenicity of inclacumab at doses up to and exceeding those previously evaluated in healthy individuals.

## Methods

### Study design

This phase 1, open-label, single-ascending-dose study of inclacumab in healthy participants was conducted at a single clinical facility (Linear Clinical Research, Nedlands, Western Australia) between September 2020 and May 2021 (Clinical Trial Registry: ACTRN12620001156976). All participants provided written informed consent before the commencement of any study**‐**related procedures. This study was conducted in full compliance with the protocol, the Declaration of Helsinki, the International Council for Harmonisation of Technical Requirements for Pharmaceuticals for Human Use and Good Clinical Practice guidelines, Therapeutic Goods Administration regulations, and all other applicable local laws and regulations. The study protocol and any amendments to the protocol were reviewed and approved by the clinical facility’s Human Research Ethics Committee.

### Participants

Healthy participants aged 18 to 65 years with a body weight between 45 and 110 kg were eligible for inclusion in this study. Participants were required to be in good health, as determined by the investigator review of medical history, physical examination, vital sign measurements, 12-lead electrocardiogram, and clinical laboratory tests.

Key exclusion criteria included hemoglobin (Hb) levels < 12 g/dL, use of prescription drugs or herbal remedies within 14 days of day –1 (except for hormonal contraceptives and some other medications at the discretion of the investigator and/or sponsor), and the use of over-the-counter medications within 7 days of day –1. Medications such as paracetamol and nonsteroidal anti-inflammatory drugs and routinely taken dietary supplements were allowed at the discretion of the investigator and/or sponsor.

### Procedures

**Fig. 1:**
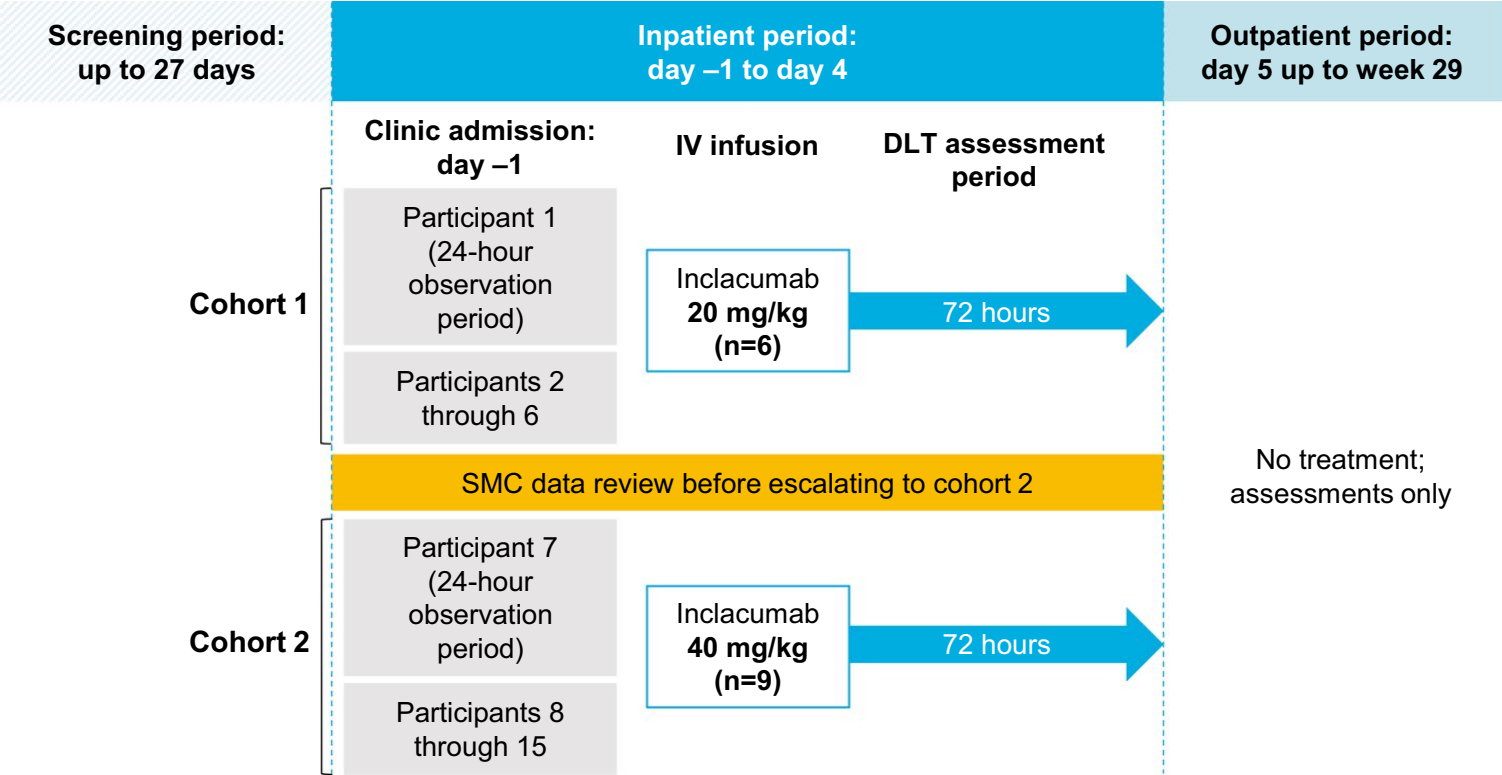
Study design. DLT, dose-limiting toxicity; IV, intravenous; SMC, safety monitoring committee

Each participant was involved in the study for up to 33 weeks, including a 27-day screening visit period (day –28 to day –2), a 4-day confinement period (day –1 to day 4), and an outpatient assessment period (day 5 up to week 29) (Fig. 1). Participants who met eligibility criteria as of day –1 were sequentially enrolled into a single-dose cohort (inclacumab 20 mg/kg or 40 mg/kg) and received the assigned dose for that cohort as an IV infusion administered over approximately 1 h on day 1.

Dosing commenced with cohort 1. Participants remained at the clinical facility and were monitored for 72 h after the infusion for the occurrence of dose-limiting toxicities (defined as any grade 3 or higher adverse event (AE) considered related to inclacumab). A safety monitoring committee reviewed AE and clinical safety laboratory data from cohort 1 participants. Cohort 2 dosing started once the safety and tolerability of cohort 1 dosing were judged to have been established by the safety monitoring committee.

After the 4-day confinement period, participants returned to the clinical facility at various intervals (weeks 1, 2, 3, 4, 6, 8, 10, 12, 14, 17, 20, 23, and 26) for study visits assessing safety, PK, and PD, and for an end-of-study visit (week 29). AEs and concomitant medications were recorded throughout the study.

### Study objectives

The primary objective of this study was to assess the safety and tolerability of inclacumab after a single dose at different dose levels in healthy participants. Secondary objectives were to characterize the plasma PK of inclacumab and the effect of inclacumab on PD in healthy participants. Characterizing the anti-drug antibody (ADA) response to inclacumab and the effects of inclacumab on other biomarkers in healthy participants, as well as exploring the relationships between PK, PD, ADA, and safety, were exploratory objectives.

### Safety and tolerability assessments

Safety was assessed in all enrolled participants. Measures of safety included the assessment of the occurrence of treatment-emergent AEs (TEAEs) and serious AEs, as well as noting clinically significant changes from baseline in vital signs (blood pressure, pulse rate, body temperature, and respiratory rate), physical examinations, electrocardiograms, and laboratory assessments (hematology panel, chemistry panel, urinalysis, coagulation panel, pregnancy test, drug screen). Infusion-related reactions were characterized as adverse reactions that were judged by the investigator to be in response to the infusion. All clinically significant laboratory value abnormalities were recorded as AEs, and any abnormal values that persisted were followed up on where directed by the investigator. Whenever possible, the severity of AEs was graded using the National Cancer Institute Common Terminology Criteria for Adverse Events, version 5.0.

### Sample collection and analysis

#### Blood sample collection

Plasma samples for PK analysis of inclacumab were obtained before administration on day 1, at the end of infusion (EOI), and at approximately 2, 6, 12, 24, and 48 h after the EOI. Subsequent samples were collected at every study visit through week 29. Plasma, serum, and/or whole-blood samples for ADA, PD, and exploratory biomarkers were also collected before administration on day 1 and at selected subsequent visits through week 29.

#### Pharmacokinetics analysis

Free inclacumab plasma concentrations were measured in samples collected from participants using a Meso Scale Discovery electrochemiluminescence assay. This validated ligand-binding assay captured inclacumab in plasma using a biotin-labeled anti-inclacumab mAb bound to a blocked streptavidin plate (Meso Scale Discovery). Captured inclacumab was then detected with a SULFO-TAG-labeled anti-inclacumab mAb. The method was applicable to the quantitation of inclacumab within a nominal range of 10 to 2560 ng/mL and required a minimum 20.0 μL human plasma aliquot containing sodium citrate. Samples were frozen at approximately − 80 °C before analysis. The analysis was performed at PPD Bioanalytical Laboratory (Richmond, VA, USA).

#### Pharmacodynamics analysis

The pharmacodynamic effects of inclacumab were assessed by determination of PLA formation, measurement of P-selectin inhibition, and the measurement of free and total soluble P-selectin plasma concentrations. Measurement of PLA formation was performed in whole blood using a validated flow cytometry method. After blood collection at the clinical research center, blood samples were activated with thrombin receptor-activating peptide (TRAP) to induce P-selectin expression on the platelet surface and subsequent PLA formation. Adding TRAP ex vivo results in activation of platelets and consequent increased P-selectin cell surface expression, resulting in more-robust PLA formation [12] and allowing better observation of PLA modulation by inclacumab. The samples were then fixed and frozen for storage and transported (at − 80 °C or on dry ice) to PPD Bioanalytical Laboratory, where the analysis was performed. For analysis, samples were brought up to room temperature, washed, and stained using a V500-conjugated anti-CD45 antibody and an allophycocyanin (APC)-conjugated anti-CD61 antibody and analyzed using a BD FACSCanto II flow cytometer. CD45-positive leukocytes, which were additionally labeled with the platelet-specific CD61-APC antibody, were defined as PLA. The percentage of PLA was determined by expressing the number of leukocytes with platelet fluorescence (CD61 positive) as a percentage of total CD45-positive leukocytes.

The inhibition of the interaction between P-selectin and PSGL-1 by inclacumab was measured in clinical samples using a validated surface plasmon resonance (SPR) assay at BioAgilytix Europe GmbH (Hamburg, Germany). The effect of inclacumab was measured as percent inhibition of spiked P-selectin fused to immunoglobulin (Psel-FC) binding to glycosulfopeptide (GSnP-6), a peptide analog that structurally resembles the N-terminal domain of PSGL-1. Initially, biotinylated GSnP-6 was injected on the streptavidin chip to immobilize the peptide for analysis. Serum samples collected from participants were then injected over the GSnP-6 sensor chip in the presence of Psel-FC. The pre-dose sample for each patient was used as the donor-specific negative control (without Psel-FC) and positive controls (in the presence of Psel-FC). The percentage of P-selectin inhibition for each sample was then calculated based on the measured response units of the sample and the respective negative and positive control values for each patient. P-selectin inhibition measured using the SPR assay has a theoretical range between 0 (no inhibition) and 100% (complete inhibition). To enable interpretation, P-selectin inhibition values above 100% and below 0% were set to the boundary result value of 100% and 0%, respectively.

Soluble P-selectin is a truncated form of P-selectin that may be produced by alternative splicing, cleaved from the cell surface, or secreted from activated platelets [13, 14]. Both free (unbound to inclacumab) and total (sum of free and inclacumab-bound) soluble P-selectin were measured in this study. The ratio of free to total soluble P-selectin reflects fractional occupancy and can be used to demonstrate target engagement of inclacumab. Free and total soluble P-selectin concentrations were determined in participant plasma samples containing sodium citrate using validated enzyme-linked immunosorbent assay (ELISA) methods at PPD Bioanalytical Laboratory. To obtain free soluble P-selectin, samples were first immunodepleted on an immunoaffinity column (Sigma PROTIA ProteoPrep Immunoaffinity Albumin and IgG Depletion Kit or Cytiva Albumin and IgG Depletion SpinTrap) that depletes both albumin and IgG (including free inclacumab and inclacumab bound to soluble P-selectin) from plasma samples. Measurements for both free and total soluble P-selectin were performed using commercially available human P-selectin/CD62P ELISA kits. Specific detection of the human soluble P-selectin was achieved via sandwich ELISA, with a murine mAb specific for human P-selectin coating on the plate for capture and a polyclonal antibody against human P-selectin for detection. The ELISA had a nominal P-selectin calibration curve range of 50.0 (upper limit of quantification) to 0.195 ng/mL (lower limit of quantification) with a minimal required dilution of 1:20.

#### Anti-drug antibody analysis

Plasma samples were analyzed for antibodies binding to inclacumab, and the titer of confirmed positive samples was reported. The detection and characterization of anti-inclacumab antibodies in participant plasma was performed using a multitiered approach with a validated bridging electrochemiluminescence immunoassay at PPD Bioanalytical Laboratory.

In this assay, samples, positive controls, and a negative control were preincubated overnight with biotin-inclacumab. Any ADA present in the plasma formed a complex with the biotin-inclacumab; the complex was then bound to a streptavidin plate (Pierce). After a wash step to remove noncomplexed components, the ADAs were eluted using glycine and incubated with biotin-inclacumab and SULFO-TAG-inclacumab molecules to form a complex for detection. This complex was bound to a blocked streptavidin plate (Meso Scale Discovery) and detected by a chemiluminescent signal generated when voltage was applied. This resulting electrochemiluminescent signal was directly proportional to the amount of ADA present.

Unknown samples were analyzed in a tiered approach. The first tier, a screening assay, identified samples as “potentially positive” when their results were equal to or above the plate-specific cut point. Samples with results below the plate-specific cut point were considered negative. The second tier, a confirmatory assay, identified “potentially positive” samples as “confirmed positive” when their results were at or above the confirmatory plate-specific cut point. Confirmed positive samples were then diluted (titered) until a negative response was obtained (third tier). The sample titer was reported as the interpolated titer result for each positive sample (inclusive of minimally required dilution).

For the mAb-positive control, the sensitivity was 3.42 ng/mL for the screening assay and 10.3 ng/mL for the confirmatory assay. For the polyclonal antibody positive control, sensitivity was < 0.477 ng/mL for the screening assay and 0.900 ng/mL for the confirmatory assay. Drug tolerance was determined to be 400 μg/mL at 100, 500, and 2500 ng/mL of anti-inclacumab monoclonal and polyclonal antibodies in the screening and confirmatory assays.

This method is applicable for the detection, confirmation, and titration of anti-inclacumab antibodies from a 20-μL participant plasma aliquot containing sodium citrate. Samples were frozen at approximately − 80 °C before analysis.

#### Statistical analysis

Safety and demographic analysis data populations and outputs were produced by the Biostatistics Department of Resolutum Global using the SAS system Version 9.4 (SAS Institute, Cary, NC, USA). The statistical methods for the safety analyses were descriptive in nature, and no formal statistical comparisons were made.

Noncompartmental analysis of PK parameters was performed using the validated software, Phoenix WinNonlin v 8.3 (Certara L.P. (Pharsight), St. Louis, MO, USA). Dose-normalized PK parameters were calculated by dividing the PK parameter by the actual administered individual dose (i.e., the assigned dose in milligrams per kilogram multiplied by baseline body weight).

The ADA data were summarized by Certara, and the impacts of ADA on PK and PD were evaluated graphically using Phoenix WinNonlin v 8.3. Results from the confirmatory assay were summarized at each time point as either confirmed ADA positive or confirmed ADA negative. The overall incidence of treatment-emergent ADA was reported for each cohort as the proportion of evaluable participants found to have seroconverted during the study period.

## Results

### Participants

#### Disposition of participants

Of the 56 potential participants screened, 41 were excluded as screen failures, and 15 were enrolled into the study (cohort 1, *n* = 6; cohort 2, *n* = 9). One participant in cohort 1 did not complete the study follow-up as planned. The participant moved to a remote location and was unable to attend any visits after day 99 and was thus considered lost to follow-up. All other participants completed the study.

#### Demographic and baseline characteristics

Participant demographics and baseline characteristics are shown in Table 1. All participants were aged 22 to 52 years at the time of screening, and the mean age of participants was 33.5 years in cohort 1 and 40.3 years in cohort 2. A similar distribution of female (46.7%) and male (53.3%) participants were enrolled in the study. The majority of participants were White in race (66.7%), with 26.7% Asian and 6.7% other races; none were of Hispanic or Latino ethnicity. The mean weight of participants in cohort 2 (78.1 kg) was greater than that for cohort 1 (69.4 kg). The mean body mass index for all participants was 25.6 kg/m^2^ and was similar between cohorts. The mean baseline serum creatinine level was also similar between cohorts.

**Table 1:** Summary of demographic and other baseline characteristics in the safety population

	**Inclacumab 20 mg/kg IV (*n* = 6)**	**Inclacumab 40 mg/kg IV (*n* = 9)**	**Pooled inclacumab IV (*N* = 15)**
Age, years			
Mean (SD)	33.5 (8.41)	40.3 (9.86)	37.6 (9.63)
Median (min, max)	30.5 (25, 44)	43.0 (22, 52)	42.0 (22, 52)
Sex, *n* (%)			
Female	3 (50.0)	4 (44.4)	7 (46.7)
Male	3 (50.0)	5 (55.6)	8 (53.3)
Ethnicity, *n* (%)			
Hispanic or Latino	0	0	0
Not Hispanic or Latino	6 (100)	9 (100)	15 (100)
Race, *n* (%)			
American Indian or Alaska Native	0	0	0
Asian	2 (33.3)	2 (22.2)	4 (26.7)
White	4 (66.7)	6 (66.7)	10 (66.7)
Other	0	1 (11.1)	1 (6.70)
Baseline height, cm			
Mean (SD)	168.7 (9.93)	171.9 (10.03)	170.6 (9.77)
Median (min, max)	169.0 (155, 184)	172.0 (155, 189)	171.0 (155, 189)
Baseline weight, kg			
Mean (SD)	69.35 (4.780)	78.07 (8.758)	74.58 (8.458)
Median (min, max)	68.85 (63.7, 75.4)	79.20 (66.2, 89.3)	73.60 (63.7, 89.3)
Baseline body mass index, kg/m^2^			
Mean (SD)	24.44 (2.485)	26.40 (2.087)	25.62 (2.383)
Median (min, max)	24.02 (21.7, 28.9)	26.68 (23.4, 28.9)	25.19 (21.7, 28.9)
Baseline serum creatinine, mg/dL			
Mean (SD)	0.822 (0.2345)	0.819 (0.1640)	0.820 (0.1871)
Median (min, max)	0.740 (0.64, 1.28)	0.760 (0.63, 1.14)	0.760 (0.63, 1.28)

*IV* intravenous, *max* maximum, *min* minimum, *SD* standard deviation

### Safety

#### Extent of exposure

All participants (*N* = 15) received a single IV dose of inclacumab on day 1, as planned. The mean (minimum, maximum) total dose administered, calculated by dividing the sum of the individual total doses by the number of participants on day 1, was 1390 mg (1270, 1510 mg) and 3120 mg (2650, 3570 mg) in cohorts 1 and 2, respectively.

#### Adverse events

A summary of TEAEs is shown in Table 2. A total of 14 participants (93.3%) reported at least 1 TEAE during the study, and all TEAEs were either mild or moderate in severity. Moderate events occurred more frequently in cohort 2 than cohort 1. Urinary tract infection was the only moderate TEAE reported in cohort 1; moderate TEAEs reported in cohort 2 included dyspepsia, depression, headache, back pain, ligament sprain, muscle strain, patella fracture, and vaccination complications.

**Table 2:** Summary of TEAEs in the safety population

	**Inclacumab 20 mg/kg IV (*n* = 6)**	**Inclacumab 40 mg/kg IV (*n* = 9)**	**Pooled inclacumab IV (*N* = 15)**
Participants with at least 1 TEAE, *n* (%) E	5 (83.3) 9	9 (100) 33	14 (93.3) 42
Mild	5 (83.3) 8	9 (100) 23	14 (93.3) 31
Moderate	1 (16.7) 1	5 (55.6) 10	6 (40.0) 11
Severe	0	0	0
Life-threatening	0	0	0
Fatal	0	0	0
Participants with at least 1 serious TEAE, *n* (%) E	0	0	0
Participants with at least 1 treatment-related TEAE, *n* (%) E	1 (16.7) 2	0	1 (6.7) 2
Participants with at least 1 dose-limiting toxicity, *n* (%) E	0	0	0
TEAEs reported by at least 2 participants overall, by preferred term, *n* (%) E			
Headache	2 (33.3) 3	4 (44.4) 6	6 (40.0) 9
Upper respiratory tract infection	0	4 (44.4) 5	4 (26.7) 5
Myalgia	0	3 (33.3) 3	3 (20.0) 3
Dermatitis contact	1 (16.7) 1	2 (22.2) 2	3 (20.0) 3
Back pain	0	2 (22.2) 2	2 (13.3) 2

*E* number of adverse events, *IV* intravenous, *TEAE* treatment-emergent adverse event

The only TEAEs assessed as potentially related to inclacumab were headache and dizziness, which were experienced by a single participant in cohort 1. Both events were mild in severity (grade 1) and resolved at the same time on day 1 after lasting for approximately 2 h. There were no dose-limiting toxicities, including grade 3 or higher infusion-related reactions, or TEAEs that led to discontinuation of inclacumab or withdrawal from the study. Headache was the only TEAE reported by 2 or more participants in cohort 1. In cohort 2, TEAEs reported by 2 or more participants were headache, upper respiratory tract infection, myalgia, contact dermatitis, and back pain.

#### Clinical laboratory evaluation

There were no notable trends in clinical laboratory findings reported during the study. At day 57, 3 participants (20.0%) showed a decrease in calcium, and at day 85, 3 participants (20.0%) showed an increase in mean corpuscular Hb concentration. These trends were not noted at any other visits, suggesting that they were transient shifts in these participants. There were no trends in coagulation or urinalysis shifts from baseline noted for the pooled data set.

Two clinically significant laboratory results (low ferritin and high transferrin) were observed, both in the same participant in cohort 2, at day 183. These out-of-range findings were consistent with the TEAE of iron-deficiency anemia that was captured for this participant on day 30 and was ongoing at the time of database lock. This TEAE was deemed by the investigator to be unrelated to inclacumab.

#### Vital signs, physical findings, and other observations related to safety

Review of the individual participant changes in vital signs and electrocardiograms by the investigator did not reveal any clinically significant individual findings or notable changes over time. Concomitant medications used during the study included oral contraceptives, ongoing vitamin use for general well-being, and medications used to treat AEs, including paracetamol and ibuprofen.

### Pharmacokinetics

#### Inclacumab plasma concentration-time profiles

**Fig. 2:**
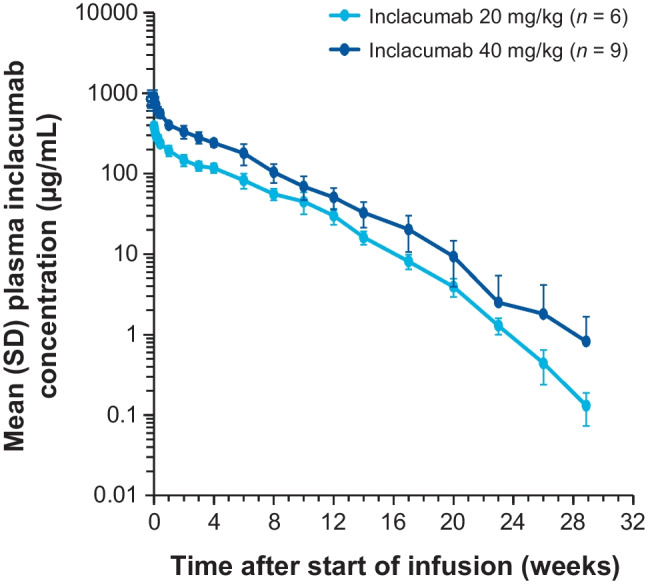
Mean (SD) plasma inclacumab concentrations over time. IV, intravenous; SD, standard deviation

Mean plasma inclacumab concentration-time profiles showed maximum concentration at or shortly after the EOI, followed by a multiphasic decline (Fig. 2). Individual concentration-time profiles generally showed a nonlinear decline toward the end of the profile, when concentrations fell below 10 µg/mL (visually approximated), consistent with target-mediated drug disposition often observed for mAbs binding to a membrane-bound target.

#### Inclacumab PK parameters

PK parameters are summarized in Table 3. After a single IV infusion of inclacumab 20 mg/kg or 40 mg/kg in healthy participants, the median time to reach maximum plasma concentration was 2.01 h and 1.03 h from the start of the approximately 1-h infusion, respectively. The area under the concentration-time curve (AUC) from time 0 to 12 weeks, AUC from time 0 to infinity, and maximum observed plasma concentration were approximately dose-proportional from 20 to 40 mg/kg. The mean (SD) concentration at week 12 was 27.9 μg/mL (5.62 μg/mL) for cohort 1 and 50.8 μg/mL (15.2 μg/mL) for cohort 2. Terminal elimination half-life (*t*_1/2_) was comparable between doses, with a mean (SD) of 13.3 days (1.66 days) for cohort 1 and 16.8 days (7.60 days) for cohort 2.

**Table 3:** Summary of plasma inclacumab PK parameters in the PK population

**PK parameter**	**Inclacumab 20 mg/kg IV (*n*** **= 6)**	**Inclacumab 40 mg/kg IV (*n*** **= 9)**
*T*_max_,^a^ h^b^	2.01 (1.00, 7.02)	1.03 (1.00, 25.0)
*C*_max_, μg/mL^c^	404 (41.4)	984 (174)
Dose-normalized *C*_max_, μg/mL/mg^c^	0.292 (0.0279)	0.317 (0.0589)
*t*_1/2_, day^c^	13.3 (1.66)	16.8 (7.60)
*AUC*_0-inf_, day·μg/mL^c^	9000 (1280)	19,300 (2760)
Dose-normalized *AUC*_0-inf_, day·μg/mL/mg^c^	6.52 (1.10)	6.22 (1.07)
*C*_12w_, μg/mL^c^	27.9 (5.62)	50.8 (15.2)

*AUC*_*0-inf*_ area under the concentration-time curve from time 0 to infinity, *C*_*max*_ maximum observed plasma concentration, *C*_*12w*_ concentration at week 12, *IV* intravenous, *min* minimum, *max* maximum, *n* number of observations, *N* number of participants, *PK* pharmacokinetics, *SD* standard deviation, *T*_*max*_ time to reach maximum plasma concentration, *t*_*1/2*_ elimination half-life

^a^From start of infusion

^b^Data are median (min, max)

^c^Data are mean (SD)

### Pharmacodynamics

#### PLA formation

**Fig. 3:**
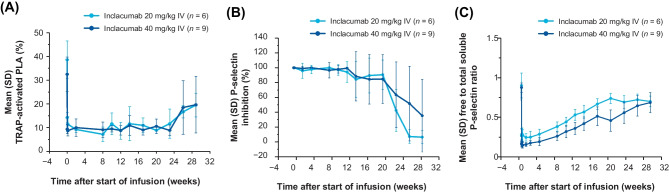
**a** Mean (SD) TRAP-activated PLA over time profiles by cohort. Time after start of infusion is based on nominal times. Pre-dose times are set to 0. Number of observations per time point varied between 3 and 6 for the 20 mg/kg dose cohort and 4 and 9 for the 40 mg/kg dose cohort. **b** Mean (SD) adjusted P-selectin inhibition over time profiles by cohort in the PD population. Time after start of infusion is based on nominal times. **c** Mean (SD) ratio of free to total soluble P-selectin over time by cohort in the PD population. Time after start of infusion is based on nominal times. IV, intravenous; PD, pharmacodynamics; PLA, platelet-leukocyte aggregate; TRAP, thrombin receptor–activating peptide

The mean (range) TRAP-activated baseline pre-dose PLA formation was 39% (29–47%) and 33% (19–46%) for cohorts 1 and 2, respectively. After administration of a single IV dose of inclacumab, mean TRAP-activated PLA formation decreased rapidly to 14% (11–16%) for cohort 1 and 9% (6–11%) for cohort 2 within 3 h from the start of the approximately 1-h infusion (Fig. 3a). Reductions in TRAP-activated PLA formation relative to baseline were sustained for both cohorts over 23 weeks, with no apparent dose dependency. Mean TRAP-activated PLA formation began to return to baseline values after week 23 but did not fully return to baseline values by 29 weeks post-dose. The mean TRAP-activated PLA formation through week 23 ranged between 7 and 12% in cohort 1, and 9 and 11% in cohort 2.

#### Surface plasmon resonance P-selectin inhibition

Serum samples collected and analyzed after administration of a single IV dose of inclacumab showed that mean P-selectin inhibition was nearly complete (defined as > 90%) for up to 12 weeks post-dose in both cohorts with a mean (range) of P-selectin inhibition at week 12 of 94.2% (65.4–100%) in cohort 1 and 99.4% (96.3–100%) in cohort 2. Subsequently, mean P-selectin inhibition appeared to decrease gradually in both cohorts. At week 29, mean (range) P-selectin inhibition was 6.38% (0–17.8%) in cohort 1 and 35.4% (0–100%) in cohort 2 (Fig. 3b).

#### Plasma soluble P-selectin

Baseline total soluble P-selectin ranged from 31.1 to 54.3 ng/mL across participants. The baseline free to total soluble P-selectin ratio ranged from 0.770 to 1.182 in cohort 1 and 0.768 to 0.965 in cohort 2. The mean free to total soluble P-selectin ratio decreased rapidly from 0.902 pre-dose to 0.266 at EOI in cohort 1, and from 0.876 pre-dose to 0.166 at EOI in cohort 2, and then generally increased gradually to 78% of baseline by the last assessment at week 29 in both cohorts (Fig. 3c).

### Anti-drug antibodies

No ADAs were observed pre-dose in any participant. Treatment-emergent ADAs were observed in 2 of 15 participants (13% overall; 0 of 6 (0%) in cohort 1 and 2 of 9 (22%) in cohort 2). For both of these participants, ADA was first detectable at week 12 and persisted through week 29. Titers remained low and within ± 2.5-fold of the titer at the day of first ADA positivity at week 12, indicating stable immunogenicity response. No clear impact of treatment-emergent ADA on the PK and PD versus time profiles was observed, and no unique safety concerns were observed in the 2 ADA-positive participants.

## Discussion

Inclacumab is a fully human IgG4 mAb directed against P-selectin being developed for treatment of patients with SCD [9, 15, 16]. Similar to previous clinical studies encompassing hundreds of participants receiving inclacumab doses up to 20 mg/kg, a single IV infusion of either 20 mg/kg or 40 mg/kg of inclacumab in healthy participants was found to be safe and well tolerated in this phase 1, open-label, single-ascending-dose study [11, 15, 17]. A higher percentage of participants in the 40 mg/kg dose cohort reported moderate TEAEs during the study; however, none were considered potentially related to inclacumab.

Inclacumab PK parameters (*C*_max_, *AUC*_0-12w_, and *AUC*_0-inf_) were approximately dose proportional from 20 to 40 mg/kg, and clearance was generally consistent across the dose range. Individual inclacumab concentration-time profiles showed a nonlinear decline toward the end of the profile when concentrations fell below 10 μg/mL, suggestive of target-mediated drug disposition and consistent with prior studies of inclacumab in healthy individuals [11, 15]. The mean terminal *t*_1/2_ was 13.3 days for the 20 mg/kg cohort and 16.8 days for the 40 mg/kg cohort; however, these reported *t*_1/2_ values and any comparison to crizanlizumab and/or other inclacumab studies should be interpreted with caution, as the *t*_1/2_ is method- and sampling-specific, particularly in a nonlinear system due to target-mediated drug disposition.

In this study, a single IV dose of inclacumab resulted in nearly complete SPR P-selectin inhibition (> 90%) up to 12 weeks post-dose in most participants in both dose cohorts. The SPR assay is an ex vivo assay that measures the inhibition of the interaction between P-selectin and its ligand PSGL-1 by inclacumab [2]. It is unknown whether ex-vivo P-selectin inhibition correlates directly with in vivo P-selectin inhibition.

To allow modulation of PLA formation by inclacumab to be observed in healthy participants, ex vivo TRAP activation was used to promote formation of PLA. Reductions in TRAP-activated PLA formation by inclacumab were sustained through approximately 23 weeks for both dose cohorts, supporting a 12-week dosing interval for future development. The mean TRAP-activated PLA formation started to return to baseline values after week 23 but did not fully return to baseline values by 29 weeks post-dose. Similar to SPR P-selectin inhibition, ex vivo inhibition of PLA formation may not directly correlate with the in vivo biology of inclacumab. However, because this marker directly interrogates cell-cell interactions, TRAP-activated PLA formation may be a better biomarker for the pharmacodynamic activity of P-selectin inhibitors in patients with SCD.

Soluble P-selectin is derived either through shedding from the cell surface or from an alternatively spliced mRNA [13, 18]. The baseline total soluble P-selectin measured here in healthy participants ranged from 31.1 to 54.3 ng/mL, consistent with other reports in healthy individuals [19]. There have been reports of increased soluble P-selectin in patients with SCD relative to levels in healthy individuals, and further increased levels during VOC. Several explanations for this have been proposed, including that the increase in soluble P-selectin is a result of endothelial dysfunction and an indicator of endothelial cell activation, or that soluble P-selectin levels in patients reflect platelet activation rather than endothelial dysfunction.

Administration of a single IV dose of inclacumab caused a rapid decrease of the free to total soluble P-selectin ratio, indicating target engagement. After the initial rapid decrease, the free to total soluble P-selectin ratio steadily increased over the study period to approximately 78% of baseline by week 29, indicating some degree of sustained target engagement for at least 7 months.

ADAs were detected in 13% of participants in this study but were not associated with a safety risk and did not appear to affect the PK or PD. In prior inclacumab studies with healthy participants, ADA incidence ranged from 4 to 10% across populations and dosing regimens [11, 15]. However, comparison of ADA incidence with historic inclacumab or other anti-P-selectin mAb data may be misleading due to differences in assay design elements (e.g., sensitivity, specificity). As no participants were ADA positive at baseline, treatment-boosted ADAs could not be evaluated in this study.

## Conclusions

In conclusion, inclacumab was well tolerated in healthy adult participants at doses up to 40 mg/kg and showed generally dose-proportional PK in the dose range tested. Durability of PD and target engagement effects through at least 12 weeks were observed after a single dose, showing the potential for a prolonged dosing interval. This study provides the foundation for continued clinical development of inclacumab in SCD [20, 21].

## Data Availability

Upon request, and subject to review, Pfizer will provide the data that support the findings of this study. Subject to certain criteria, conditions and exceptions, Pfizer may also provide access to the related individual de-identified participant data. See https://www.pfizer.com/science/clinical-trials/trial-data-and-results for more information.
